# Assessing the Co-Exposure Patterns of Volatile Organic Compounds and the Risk of Hyperuricemia: An Analysis of the National Health and Nutrition Examination Survey 2003–2012

**DOI:** 10.3390/toxics12110772

**Published:** 2024-10-24

**Authors:** Xue Zhao, Haiyan Ding, Jian Qin, Shuli An, Shuangshuang Li, Hongqi He, Linwei Zhou, Xinjie Gong, Xia Chu

**Affiliations:** Department of Nutrition and Food Hygiene, College of Public Health, Key Laboratory of Precision Nutrition and Health, Ministry of Education, Harbin Medical University, No. 157 Baojian Road, Nangang District, Harbin 150081, China; 202201064@hrbmu.edu.cn (X.Z.);

**Keywords:** volatile organic compounds, co-exposure, hyperuricemia, combined effects, K-means clustering

## Abstract

Background: Co-exposure to multiple volatile organic compounds (VOCs) is common in daily life. However, few studies have evaluated the associations between the patterns of simultaneous exposure to multiple VOCs and the risk of hyperuricemia. Methods: This study included 7490 adults from the National Health and Nutrition Examination Survey conducted between 2003 and 2012. The K-means clustering method was applied to cluster eight kinds of VOCs in the blood into various co-exposure patterns, including benzene, bromodichloromethane, chloroform, dibromochloromethane, 1,4-dichlorobenzene, ethylbenzene, methyl tertiary-butyl ether (MTBE), and o-xylene. Binary logistic regression analysis was utilized to assess the association between single VOCs, the co-exposure patterns of multiple VOCs, and the hyperuricemia risk. Restricted cubic spline functions were utilized to investigate the non-linear relationships. Results: Based on eight VOCs, four characteristic co-exposure patterns were generated. Compared with the low-level exposure group, the levels of volatile organic compound (VOC) co-exposure in cluster 2, characterized by relatively high levels of MTBE and moderate levels of bromodichloromethane, chloroform, and dibromochloromethane, were associated with increased hyperuricemia risk, with an odds ratio of 1.32 (1.02, 1.71). Increasing levels of bromodichloromethane and chloroform were significantly associated with an increased risk of hyperuricemia. A strong J-shaped relationship was found between MTBE and hyperuricemia. Conclusions: This study indicated that blood bromodichloromethane and chloroform were positively associated with hyperuricemia risk. Blood MTBE had a J-shaped association with hyperuricemia. In addition, the significant association of the co-exposure patterns of multiple VOCs in the blood with hyperuricemia risk was observed. Changing VOC co-exposure patterns may play a crucial role in the occurrence of hyperuricemia.

## 1. Introduction

Hyperuricemia, which has become an important public health problem, is caused by either the overproduction or underexcretion of uric acid (UA) [1]. Current research has extensively shown that hyperuricemia not only serves as a critical precursor to developing gout but also stands as an independent risk factor for diseases, including hypertension, cardiovascular diseases, and metabolic syndrome [2,3,4]. The significant prevalence of these diseases related to hyperuricemia profoundly affects quality of life and contributes substantially to the overall societal burden of disease.

Volatile organic compounds (VOCs) encompass a broad array of chemicals commonly utilized in industries and consumer products as solvents, degreasers, and cleaning agents [5]. These compounds pervade daily life through various sources, including smoking, cleaning supplies, building materials, vehicle emissions, and industrial processes [6,7]. The primary routes of human exposure to VOCs are inhalation and dietary intake. Prolonged exposure to these compounds can lead to adverse health effects on the respiratory system, nervous system, and digestive system [8,9,10]. Increasing evidence has revealed the VOCs’ accumulating toxicity [11]. Mikulski et al. found that exposure to toluene and xylene among ship painters could impair the excretion of UA [12]. An experimental study in rats revealed that depurinized milk with a reduced volatile organic compound (VOC) concentration shows numerous beneficial effects, which may be recommended for the nutritional treatment of primary or secondary hyperuricemia [13]. However, Ahmad et al. have reported that serum uric acid (SUA) showed a significant decrease in rats exposed to benzene consecutively for 10 days [14]. The current studies indicate that the specific impacts of VOCs on UA levels remain poorly understood, and no studies have previously explored the relationship between VOC exposure and the risk of hyperuricemia.

Co-exposure to multiple VOCs in daily life can result in complex interactions in the body. Therefore, it is essential to assess the impact of VOC co-exposure on hyperuricemia. The concentration of VOCs in the blood effectively reflects the body’s exposure to these compounds [15]. Therefore, we explored the relationship between eight kinds of VOCs in the blood and hyperuricemia with the National Health and Nutrition Examination Survey (NHANES) conducted between 2003 and 2012. The K-means clustering method was applied to cluster eight kinds of VOCs in the blood into various patterns of VOC co-exposure. Multivariate logistic regression analysis was utilized to evaluate the association between single VOCs, the co-exposure patterns of multiple VOCs, and the risk of hyperuricemia. Restricted cubic spline (RCS) functions were utilized to investigate the non-linear relationships. In addition, stratification analyses were conducted to elucidate the effects of VOCs across a representative sample of various subgroups.

## 2. Methods

### 2.1. Study Design and Participants

The NHANES is a representative multistage study conducted to access health-related data on the non-institutionalized civilian population of the United States [16]. The researchers collect data via household interviews and perform physical examinations and biological specimen collection at mobile examination centers. The survey has been approved by the Research Ethics Review Board at the National Center for Health Statistics. In addition, written informed consent is obtained from all participants, ensuring compliance with ethical standards.

**Figure 1:**
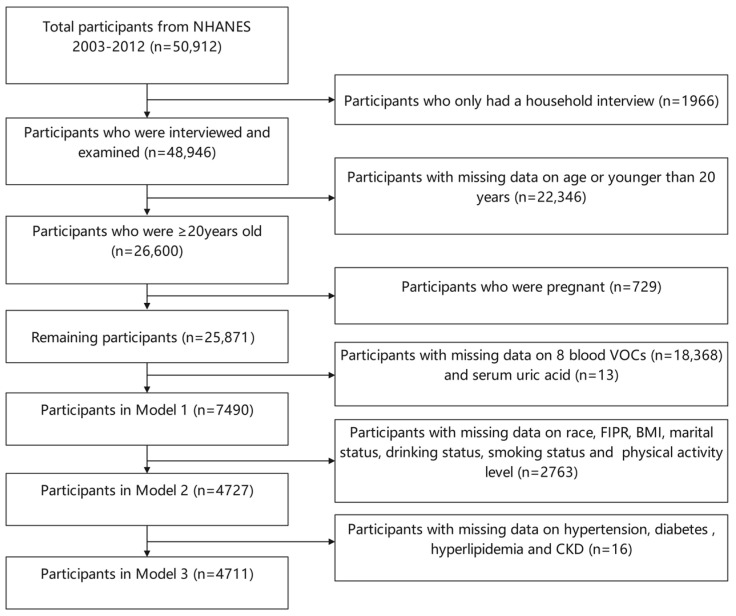
Flowchart for the selection of participants in this study. FIPR, family income-to-poverty ratio; BMI, body mass index; CKD, chronic kidney disease.

In the current study, we utilized data from five 2-year cycles of the NHANES conducted between 2003 and 2012. There were 50,912 participants in the NHANES from 2003 to 2012, and 26,600 participants aged over 20 years old were included in this analysis. After excluding individuals who were pregnant (*n* = 729) and participants with missing information on 8 blood VOCs (*n* = 18,368) and SUA (*n* = 13), our statistical analysis included 7490 participants with complete measurements of blood VOC and SUA concentrations (Figure 1).

### 2.2. Measurement of Blood VOCs and Serum Uric Acid

In this study, eight common VOCs were analyzed, including benzene, bromodichloromethane, chloroform, dibromochloromethane, 1,4-dichlorobenzene, ethylbenzene, methyl tertiary-butyl ether (MTBE), and o-xylene. The levels of VOCs in the blood were accurately measured by solid-phase microextraction/gas chromatography/mass spectrometry. Information on the laboratory measurements can be found on the NHANES website (Supplementary Table S1).

Laboratory technicians utilized a timed endpoint method to measure the SUA concentration. Hyperuricemia is defined as an SUA level of ≥420 μmol/L (7 mg/dL) in males and ≥360 μmol/L (6 mg/dL) in females [1].

### 2.3. Assessment of Covariates

Covariates included gender, age, race/ethnicity, family income-to-poverty ratio (FIPR), body mass index (BMI), marital status, drinking status, smoking status, physical activity level, hypertension, diabetes, hyperlipidemia, and chronic kidney disease (CKD). The races/ethnicities consisted of non-Hispanic White, non-Hispanic Black, Mexican American, other Hispanic, or other races. The FIPR was classified as <1.0 and ≥1.0. The BMI was calculated by dividing weight in kilograms by height in meters squared. Marital status included married, living with partner, widowed, divorced, separated, or never married. Drinking status was grouped into never drinker (<12 alcohol drinks in lifetime), former drinker (≥12 alcohol drinks in lifetime but quit drinking last year), mild drinker (≥1 alcohol drink for females or ≥2 alcohol drinks for males per day), moderate drinker (≥2 alcohol drinks for females per day, ≥3 alcohol drinks for males per day, or binge drinking ≥2 and <5 days per month), or heavy drinker (≥3 alcohol drinks for females per day, ≥4 alcohol drinks for males per day, or binge drinking ≥5 days per month). Binge drinking was defined as consuming ≥4 alcohol drinks for females or ≥5 alcohol drinks for males on same occasion. Smoking status was classified as never smoker (<100 cigarettes in lifetime), former smoker (≥100 cigarettes in lifetime but quit smoking now), or current smoker (≥100 cigarettes in lifetime and currently smoke cigarettes every day). The physical activity level was categorized as high (>1000 metabolic equivalent MET-min per week), moderate (500–1000 MET-min per week), or insufficient (<500 MET-min per week). Hypertension was defined as a systolic pressure ≥140 mmHg, a diastolic pressure ≥90 mmHg, self-reported physician’s diagnosis of hypertension, or self-reported use of anti-hypertension medications. Diabetes was defined as self-reported physician’s diagnosis of the disease or the use of antidiabetic medications. Hyperlipidemia referred to the diagnosis of hypercholesterolemia or hypertriglyceridemia or the use of lipid-lowering medications. CKD was defined as an estimated glomerular filtration rate (eGFR) < 60 mL/min/1.73 m^2^ and/or albuminuria, which was indicated by an albumin-to-creatinine ratio (ACR) ≥ 30 mg/g [17]. The eGFR was calculated employing the Chronic Kidney Disease Epidemiology Collaboration formula [18].

### 2.4. Statistical Analysis

The NHANES weights and strata variables were considered when calculating statistics. To assess differences in population characteristics within survey cycles and cluster groups, we employed the Rao–Scott chi-square test for categorical variables and the Kruskal–Wallis test for continuous variables. We reported medians and the interquartile range (IQR) for continuous variables and percentages for categorical variables. Prior to analysis, the concentrations of the eight blood VOCs were log-transformed and then standardized to eliminate the influence of different units. The K-means clustering method was performed to classify eight blood VOCs into distinct groups. We considered the elbow method and the eigenvalues of the covariance matrix of VOCs to identify the number of clusters. We conducted three logistic regression models to examine the association between different VOC clusters and hyperuricemia and calculated odds ratios (ORs) and their corresponding 95% confidence intervals (CIs) for each model. Model 1 was adjusted for gender and age. Model 2 was additionally adjusted for race, FIPR, BMI, marital status, drinking status, smoking status, and physical activity level. Model 3 was additionally adjusted for hypertension, diabetes, hyperlipidemia, and CKD. Stratified analyses were performed based on gender, age (<60 years or ≥60 years), and BMI (<25 kg/m^2^ or ≥25 kg/m^2^), with potential modifying effects assessed through multiplicative interaction terms. 

Binary logistic regression analysis was conducted to examine the relationship between single blood VOCs and hyperuricemia using the R package “survey”. Considering the potential non-linear relationship, RCS analysis was applied with three knots located at the 10th, 50th, and 90th percentiles in a fully adjusted model using the “rms” package of R. The significance of the overall and non-linear associations between the eight VOCs and hyperuricemia was determined using an ANOVA test in the R package “car”.

To verify the robustness of our findings, we conducted two sensitivity analyses. First, considering that the use of certain medications, such as furosemide, losartan, and allopurinol, can impact UA metabolism, we reperformed the analyses excluding participants with medication use. In addition, all models were rerun after excluding participants with at least three of the four chronic diseases (hypertension, diabetes, hyperlipidemia, or CKD).

A *p* value of <0.05 was considered statistically significant for two-sided tests. All analyses were performed using R version 4.3.2. 

## 3. Results

### 3.1. Cluster Analysis of Blood VOCs

According to the eigenvalues of the covariance matrix of VOCs and the evaluation criteria for clustering in Supplementary Table S2 and Supplementary Figure S1, eight blood VOCs in 7490 participants were clustered into four groups. Table 1 presents the center characteristics of the four clusters for the studied VOCs. With respect to the four clusters, based on the centers of the four clusters of the blood VOCs and the quartile distributions of standardized VOC concentrations (Supplementary Table S3), we defined cluster 1 as the reference for subsequent analysis because the eight blood VOC concentrations in cluster 1 were relatively low compared with the other three clusters. Cluster 2 was characterized by comparatively high levels of MTBE above the 75th percentile and moderate levels of bromodichloromethane, chloroform, and dibromochloromethane close to or above the median of their respective distributions. Cluster 3 had relatively higher levels of benzene, ethylbenzene, and o-xylene because their concentrations were above their 75th percentiles. Cluster 4 had relatively higher levels of bromodichloromethane, chloroform, and dibromochloromethane because their concentrations were close to or above their 75th percentiles in the blood. The quartile distributions of unadjusted VOC concentrations also illustrated the characteristics of the eight blood VOCs in the four clusters, as shown in Supplementary Table S4. 

### 3.2. Baseline Characteristics of All Participants

Supplementary Table S5 and Table 2 present the baseline characteristics of all participants from the five cycles and those in the four clusters, respectively. The covariates of gender, race, FIPR, BMI, marital status, drinking status, smoking status, physical activity level, and diabetes were unevenly distributed among the clusters. Specifically, more participants in cluster 1 were mild drinkers and never smokers and had a high physical activity level compared to the other three clusters. Cluster 3 predominantly consisted of males, heavy drinkers, current smokers, and participants with a low FIPR. The proportions of non-Hispanic White people were larger in all clusters compared to other ethnic groups.

### 3.3. Association between Single Blood VOCs and the Risk of Hyperuricemia

The results of the multiple logistic regression analysis exploring the relationship between single blood VOCs and the risk of hyperuricemia are presented in Table 3. An increase of ten units in the natural log-transformed levels of bromodichloromethane and chloroform was related to an increased risk of hyperuricemia. The ORs (95% CIs) of hyperuricemia were 2.56 (1.10, 5.96) for bromodichloromethane and 2.13 (1.01, 4.53) for chloroform in Model 3. 

**Figure 2:**
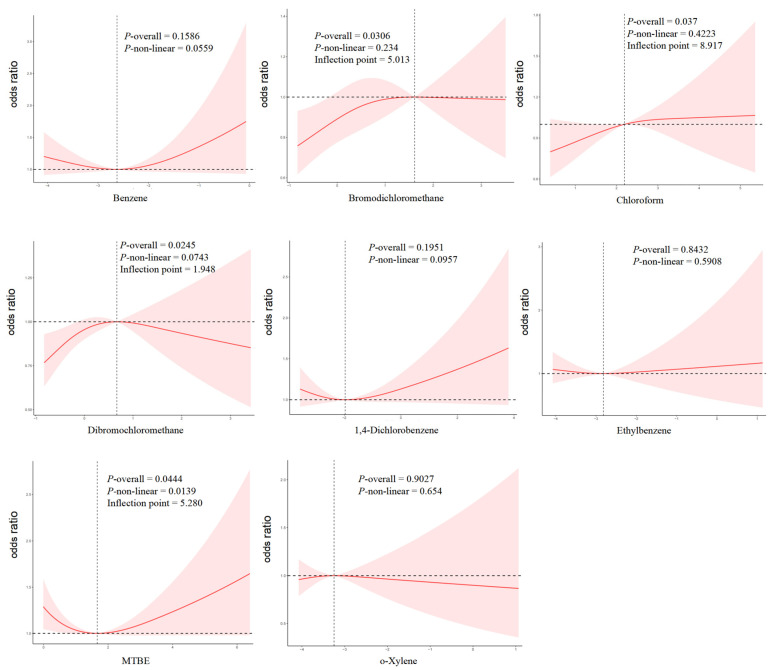
Adjusted exposure–response relationship between concentrations of each blood VOC and the risk of hyperuricemia. VOCs were coded using the RCS function with three knots located at the 10th, 50th, and 90th percentiles. The *X*-axis represents the ln-transformed concentration of blood VOCs. The *Y*-axis represents the adjusted odds ratios of hyperuricemia risk based on the value of blood VOC levels. The inflection point shows the original concentration of blood VOCs. All models were adjusted for gender, age, race, FIPR, BMI, marital status, drinking status, smoking status, physical activity level, hypertension, diabetes, hyperlipidemia, and CKD. FIPR, family income-to-poverty ratio; BMI, body mass index; CKD, chronic kidney disease.

The RCS analysis revealed that the levels of bromodichloromethane, chloroform, dibromochloromethane, and MTBE were associated with the risk of hyperuricemia in Model 3 (*p* overall < 0.05) (Figure 2). The highest risk of hyperuricemia was observed at blood concentrations of 5.013 pg/mL for bromodichloromethane and 1.948 pg/mL for dibromochloromethane. With an elevated blood concentration of chloroform, the risk of hyperuricemia increased rapidly and tended to increase slowly when the concentration reached 8.917 pg/mL. A significant non-linear exposure–response relationship was identified between MTBE and the risk of hyperuricemia (*p* non-linear < 0.05), characterized by a J-shaped curve. When the concentrations of MTBE were close to 5.28 pg/mL, the risk of hyperuricemia reached a relatively low level.

### 3.4. Multiple VOC Co-Exposure and the Risk of Hyperuricemia

**Figure 3:**
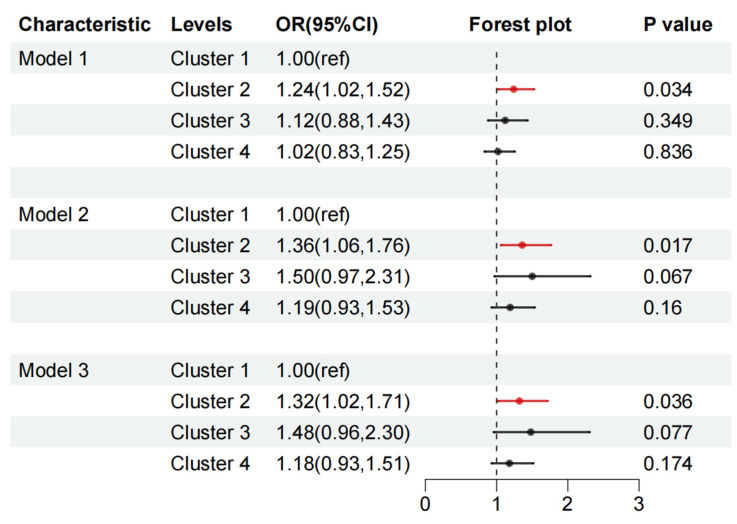
Odds ratios (95% CIs) of hyperuricemia in relation to multiple VOC co-exposure clusters among participants. Model 1 was adjusted for gender and age. Model 2 was additionally adjusted for race, FIPR, BMI, marital status, drinking status, smoking status, and physical activity level. Model 3 was additionally adjusted for hypertension, diabetes, hyperlipidemia, and CKD. FIPR, family income-to-poverty ratio; BMI, body mass index; CKD, chronic kidney disease. The red lines represent *p* value <0.05 and the black lines represent *p* value >0.05.

To assess the combined effects of multiple VOC co-exposure on the hyperuricemia risk, multi-covariate-adjusted logistic regression models were performed. The forest plots in Figure 3 illustrate the correlation between the patterns of VOC clustering and hyperuricemia risk. Compared with cluster 1, the OR (95% CIs) of hyperuricemia in cluster 2 was 1.36 (1.06, 1.76) after adjusting for gender, age, race, FIPR, BMI, marital status, drinking status, smoking status, and physical activity level in Model 2. When further adjusting for hypertension, diabetes, hyperlipidemia, and CKD, the OR (95% CIs) of hyperuricemia in cluster 2 was 1.32 (1.02, 1.71) in Model 3.

### 3.5. Subgroup Analysis 

Stratified analyses were conducted to explore if the effects of VOC co-exposure on hyperuricemia risk differed across subgroups defined by gender, age, and BMI. As presented in Supplementary Figure S2, the association between cluster 2 and the increased risk of hyperuricemia remained consistent in all subgroups after adjusting for all covariates. Interestingly, age appeared to modify the relationship between blood VOC levels and hyperuricemia risk significantly (*p* for interaction = 0.012). However, no significant interactions were observed for subgroups stratified by gender or BMI. 

### 3.6. Sensitivity Analysis

Supplementary Table S6 and Table S7 show the sensitivity analysis results. When we excluded participants with medication use that could potentially affect UA metabolism, cluster 2 was still associated with the risk of hyperuricemia (Supplementary Table S6), with an OR (95% CIs) of 1.45 (1.09, 1.92). In the fully adjusted model, the results did not change significantly when we excluded participants with at least three of the four chronic diseases (hypertension, diabetes, hyperlipidemia, or CKD) (Supplementary Table S7), with an OR (95% CIs) of 1.37 (1.02, 1.85).

## 4. Discussion

Exposure to VOCs is linked to adverse effects that impact various systems in the body, including the respiratory system [9], nervous system [10], and digestive system [8], but their associations with hyperuricemia remain unclear and warrant comprehensive research. Therefore, this study explored the impact of single VOCs and the co-exposure patterns of multiple VOCs on the risk of hyperuricemia. In this study, increasing levels of bromodichloromethane and chloroform were associated with a high risk of hyperuricemia. A strong J-shaped relationship was found between MTBE and hyperuricemia. In addition, using the K-means clustering method, eight kinds of VOCs in the blood, including benzene, bromodichloromethane, chloroform, dibromochloromethane, 1,4-dichlorobenzene, ethylbenzene, MTBE, and o-xylene, were grouped into four co-exposure patterns. The levels of VOC co-exposure in cluster 2, characterized by relatively high levels of MTBE and moderate levels of bromodichloromethane, chloroform, and dibromochloromethane, were associated with a significantly increased risk of developing hyperuricemia. 

Disinfection by-products (DBPs), including bromodichloromethane, chloroform, and dibromochloromethane, are formed when chlorine, used as a disinfectant, reacts with natural organic materials in water. Chlorinated drinking water and recreational water are primary sources of DBPs [19,20]. DBPs are known for their cytotoxic, mutagenic, teratogenic, and carcinogenic effects on human health [21]. MTBE was widely used as an additive to replace lead in gasoline due to its high production volume, and it can penetrate into ground water and air with high probability [22]. Despite the application of MTBE being forbidden in the US, it continues to be produced for exportation to countries where its use remains legal. Benzene, ethylbenzene, and o-xylene are commonly released into the environment through cigarette smoke, automobile exhausts, and factory emissions [23]. Dichlorobenzenes, particularly 1,4-dichlorobenzene, have been used industrially as moth repellents and deodorizers, indicating their pervasive use in chemical synthesis [24]. Occupational exposure to VOCs has been linked to increased cancer risk [25,26]. A study reported that the excretion of UA is impaired among ship painters exposed to toluene and xylene [12]. Although environmental exposure to VOCs generally occurs at lower levels than occupational exposure, a substantially larger population is affected. VOCs remain a critical environmental health concern due to their widespread use and the severe health risks they cause.

The impact of exposure to DBPs on human health has been suggested by accumulating epidemiological and animal studies. A prospective study by Sun et al. identified a positive correlation between blood bromodichloromethane levels and the risk of cancer mortality [27]. Additionally, several investigations have linked chronic chloroform exposure to colorectal and bladder cancers [28,29]. Furthermore, Burch et al. reported that exposure to bromodichloromethane was associated with elevated alanine aminotransferase activity in circulation, which is a key indicator for the early identification of non-alcoholic fatty liver disease [30]. Till now, the effect of bromodichloromethane and chloroform on hyperuricemia has not been explored. Our findings for the first time demonstrate a positive correlation between the levels of blood bromodichloromethane and chloroform and the risk of developing hyperuricemia in the population. However, the mechanism for such an association is uncertain. Two hypotheses may contribute to our results. First, bromodichloromethane and chloroform can produce toxicity through the production of reactive metabolites and oxidative stress by cytochrome P450 (CYP) activity in the liver [31,32]. An animal study showed that the metabolism of bromodichloromethane contributed to the production of toxic free radical metabolites and exacerbated inflammation in obese mice [31]. A cell experiment has reported that cell death induced by chloroform occurs in a metabolic phase where glutathione is depleted and an oxidative phase where mitochondrial permeability is transitioned and proteins are nitrated [32]. Enhancing oxidative stress may upregulate xanthine oxidase (XO) [33], a key enzyme in UA production. Thus, we hypothesize that bromodichloromethane and chloroform metabolism, by enhancing oxidative stress and upregulating XO, may lead to more synthesis of UA, affecting the concentration of UA in circulation. Second, the kidney plays a crucial role in UA reabsorption and excretion. Liu et al. reported an inverse association between bromodichloromethane, chloroform, and eGFR, which is a main measure of kidney function [34]. In a variety of animal models, repeated exposure to bromodichloromethane and chloroform has induced renal tubular degeneration, necrosis, and dysfunction [35,36]. VOCs seem to be able to affect renal dysfunction and lead to a decrease in UA excretion. However, further research is warranted to validate our speculations.

Previous studies mainly paid attention to the tumorigenic effects of MTBE [37]. In recent years, some studies have highlighted its association with metabolic disorders, such as alterations in insulin structure, the generation of reactive oxygen species, and the disorder of glucose and lipid metabolism [38,39]. However, evidence regarding MTBE’s impact on UA levels in the population remains rare. In our study, RCS analysis revealed a strong J-shaped relationship between MTBE and hyperuricemia. Some previous evidence may explain our results. First, research conducted on both cellular and animal models has consistently shown that MTBE can trigger oxidative stress [40,41,42,43]. One possible explanation is that increased oxidative stress caused by MTBE could potentially upregulate XO, resulting in increased SUA levels. Second, Bermudez et al. have reported that MTBE exposure in male rats leads to elevated kidney weights and tubular necrosis [44]. Another possible explanation is that impaired renal function caused by MTBE may result in the decreased excretion of UA, leading to a high level of UA in circulation. Although the potential mechanism of the J-shaped relationship between MTBE and hyperuricemia is still inconclusive, we speculate that oxidative stress is not induced with exposure to MTBE concentrations within a low-level range. However, exceeding a certain threshold will trigger oxidative stress, leading to increased UA production. At the same time, the compensatory protective mechanism in the body results in a slight reduction in SUA by increasing UA excretion in the kidneys. When MTBE concentrations increase beyond the body’s compensatory response, the increased SUA levels occur later. Further studies are needed to validate these hypotheses.

In this study, we identified four clustering patterns characterized by varying concentrations of VOCs to investigate the impact of different patterns of VOC composition on the risk of hyperuricemia. The fully adjusted model showed that the levels of VOC co-exposure in cluster 2, characterized by relatively high levels of MTBE and moderate levels of bromodichloromethane, chloroform, and dibromochloromethane, were associated with an increased hyperuricemia risk. This result may be attributed to the following mechanisms. One possible explanation for these results is the multi-pathway effect. Bromodichloromethane, chloroform, and MTBE induced oxidative stress in in vivo and in vitro models [31,32,40], which may affect the synthesis of UA. Exposure to bromodichloromethane, chloroform, and MTBE may impair kidney function [34,35,36,42,44], which may lead to the abnormal excretion of UA. Therefore, co-exposure to multiple VOCs can exert their effects via various pathways simultaneously, increasing the risk of hyperuricemia. Moreover, another possible explanation is a synergistic effect between VOCs. A study by Hernando et al. demonstrated that MTBE can enhance the toxicity of other pollutants present in the same matrix, indicating the existence of synergistic interactions between contaminants [45]. Additionally, Tao et al. revealed that the addition of chloroform increased the DNA hypomethylation induced by dichloroacetic acid in the kidney [46]. Thus, simultaneous exposure to multiple VOCs may lead to synergistic effects, potentially promoting their interaction. Future in-depth studies are warranted to explore and quantify the dose–response relationships between co-exposure to multiple VOCs and the risk of hyperuricemia. Furthermore, further research is required to clarify the underlying biological mechanisms of VOC interactions.

Stratified analyses revealed that there was an interaction effect of age on the relationship between VOC co-exposure and hyperuricemia risk. Although the specific mechanism is still unclear, several possible hypotheses could be considered. First, as individuals become older and more health-aware, they tend to be less exposed to VOCs. Second, as a key VOC metabolic enzyme, CYP activity greatly affects the VOC concentration in the blood. Sinues et al. demonstrated that age was an important factor affecting CYP2A6 activity, with older subjects having higher activity [47]. Therefore, it could be speculated that healthy living habits or elevated metabolic enzyme activities in the elderly reduce the concentration of VOCs in the blood. Future studies are necessary to confirm the biological mechanism of how age interacts with the effect of VOC co-exposure on hyperuricemia.

This study has several strengths. Firstly, this study uses a large national sample to examine the association between the effects of single blood VOCs and the co-exposure patterns of multiple blood VOCs generated by the K-means clustering method on hyperuricemia. Secondly, the weighted data could make the findings representative across the US population. However, our study has some limitations. Firstly, while our study encompasses a nationwide scope, the majority of the data originate from the US population. There is a lack of data for Asian or other populations, and variations in VOC exposure may arise due to differences in the developmental status of countries. Secondly, although we found a correlation between blood VOC levels and the risk of hyperuricemia, pinpointing the specific sources of these blood VOCs was challenging, constrained by the available population information. Thirdly, the half-life of VOCs is relatively short. Blood VOCs were measured only once at baseline, which did not accurately reflect long-term exposure. Repeated measurements will be made in future studies. Fourthly, despite comprehensive adjustments for covariates, some confounding factors were not fully controlled due to insufficient information on genetic factors, medication dosages, and treatment adherence. Finally, as a cross-sectional study, there remains uncertainty in determining causality between VOC exposure and hyperuricemia risk. Future longitudinal studies are warranted to confirm the causal relationships.

## 5. Conclusions

The findings of this cross-sectional study indicated that blood bromodichloromethane and chloroform were positively associated with hyperuricemia risk, and MTBE had a J-shaped association with hyperuricemia. In addition, the significant association of the co-exposure patterns of multiple VOCs in the blood with hyperuricemia risk was observed. Changing VOC co-exposure patterns may play a crucial role in the occurrence of hyperuricemia. Relevant blood VOC concentrations provide important guidance for the prevention and management of hyperuricemia in clinical practice and public health policy-making. Future mechanistic studies at the animal and cellular levels are necessary to validate the effects of VOCs on the risk of hyperuricemia in various VOC co-exposure patterns.

## Figures and Tables

**Table 1:** Centers of four clusters of the eight blood VOCs pre-processed by log transformation and standardization.

Variables	Cluster 1	Cluster 2	Cluster 3	Cluster 4
Benzene	−0.480	−0.109	1.587	−0.401
Bromodichloromethane	−0.654	−0.053	−0.124	1.123
Chloroform	−0.483	0.242	−0.040	0.615
Dibromochloromethane	−0.530	−0.318	−0.134	1.106
1,4-Dichlorobenzene	−0.136	0.414	−0.055	−0.009
Ethylbenzene	−0.530	0.065	1.563	−0.416
MTBE	−0.416	1.722	−0.125	−0.342
o-Xylene	−0.488	0.244	1.251	−0.355

**Table 2:** Baseline characteristics of participants in NHANES 2003–2012, stratified by the four clusters of blood VOCs.

Characteristics	Cluster 1	Cluster 2	Cluster 3	Cluster 4	*p* Value
*n* (%)	2924 (39.04)	1194 (15.94)	1452 (19.39)	1920 (25.63)	
Gender					<0.0001
Male	1423 (46.96)	597 (48.63)	848 (56.09)	877 (46.51)	
Female	1501 (53.04)	597 (51.37)	604 (43.91)	1043 (53.49)	
Age (years)	45 (32, 59)	44 (33, 54)	45 (34, 54)	44 (31, 57)	0.11
Race/ethnicity					<0.0001
Non-Hispanic White	1375 (69.73)	509 (65.67)	823 (75.63)	727 (63.11)	
Non-Hispanic Black	576 (10.16)	333 (14.87)	356 (12.50)	406 (11.37)	
Mexican American	427 (7.05)	255 (9.96)	120 (3.88)	418 (11.01)	
Other Hispanic	250 (5.24)	48 (3.84)	90 (3.69)	238 (7.80)	
Other race	296 (7.82)	49 (5.67)	63 (4.30)	131 (6.71)	
FIPR					<0.0001
<1.0	467 (11.47)	197 (11.24)	373 (19.11)	332 (12.13)	
≥1.0	2227 (88.53)	944 (88.76)	967 (80.89)	1448 (87.87)	
BMI (kg/m^2^)	27.80 (24.17, 32.40)	27.87 (24.20, 32.50)	26.23 (23.12, 30.60)	27.39 (23.99, 31.84)	<0.0001
Marital status					<0.0001
Married	1578 (57.95)	659 (60.43)	619 (47.42)	1028 (56.00)	
Living with partner	190 (6.61)	74 (5.91)	191 (13.67)	126 (6.20)	
Widowed	270 (6.71)	82 (4.42)	92 (4.20)	143 (4.70)	
Divorced	279 (8.77)	112 (9.19)	215 (13.27)	179 (8.92)	
Separated	84 (2.02)	32 (2.35)	65 (2.99)	74 (2.65)	
Never married	521 (17.94)	235 (17.69)	269 (18.44)	369 (21.52)	
Drinking status					<0.0001
Never drinker	364 (11.15)	178 (14.10)	73 (4.91)	278 (12.01)	
Former drinker	499 (14.35)	226 (18.05)	246 (17.01)	287 (13.74)	
Mild drinker	940 (40.42)	324 (31.55)	361 (26.01)	562 (36.83)	
Moderate drinker	367 (15.60)	153 (15.39)	228 (18.16)	256 (17.30)	
Heavy drinker	463 (18.47)	215 (20.91)	418 (33.90)	339 (20.11)	
Smoking status					<0.0001
Never smoker	1867 (64.98)	671 (55.04)	237 (16.60)	1186 (62.18)	
Former smoker	822 (27.46)	313 (28.91)	125 (8.74)	534 (27.66)	
Current smoker	233 (7.56)	210 (16.05)	1090 (74.66)	200 (10.17)	
Physical activity level					<0.0001
High	1226 (56.85)	253 (28.22)	599 (54.77)	795 (54.05)	
Moderate	375 (17.38)	208 (25.43)	183 (17.51)	212 (15.35)	
Insufficient	548 (25.77)	435 (46.35)	294 (27.72)	401 (30.60)	
Hypertension					0.89
No	1740 (65.07)	735 (64.81)	879 (66.34)	1146 (64.91)	
Yes	1183 (34.93)	459 (35.19)	573 (33.66)	772 (35.09)	
Diabetes					0.01
No	2536 (90.28)	1056 (90.54)	1317 (93.16)	1703 (92.85)	
Yes	388 (9.72)	138 (9.46)	135 (6.84)	217 (7.15)	
Hyperlipidemia					0.52
No	778 (28.10)	342 (28.91)	408 (27.94)	549 (30.35)	
Yes	2146 (71.90)	852 (71.09)	1044 (72.06)	1371 (69.65)	
CKD					0.31
No	2341 (85.89)	998 (86.88)	1206 (88.21)	1563 (87.72)	
Yes	560 (14.11)	180 (13.12)	227 (11.79)	340 (12.28)	

FIPR, family income–poverty ratio; BMI, body mass index; CKD, chronic kidney disease. *p* values were calculated using the Rao–Scott chi-square test for categorical variables and the Kruskal–Wallis test for continuous variables.

**Table 3:** Odds ratios (95% CIs) of hyperuricemia in relation to per ten increments in ln-transformed VOCs.

Variables	Model 1		Model 2		Model 3	
	OR (95%CI)	*p* Value	OR (95%CI)	*p* Value	OR (95%CI)	*p* Value
Benzene	1.11 (0.52, 2.35)	0.790	1.32 (0.21, 8.42)	0.767	1.08 (0.17, 6.96)	0.933
Bromodichloromethane	1.04 (0.50, 2.17)	0.920	2.48 (1.04, 5.90)	0.040	2.56 (1.10, 5.96)	0.031
Chloroform	1.25 (0.64, 2.44)	0.515	1.89 (0.86, 4.13)	0.111	2.13 (1.01, 4.53)	0.049
Dibromochloromethane	0.98 (0.46, 2.07)	0.950	2.38 (0.94, 6.06)	0.068	2.43 (0.94, 6.28)	0.067
1,4-Dichlorobenzene	1.68 (1.05, 2.70)	0.032	1.21 (0.53, 2.75)	0.644	1.24 (0.53, 2.92)	0.613
Ethylbenzene	0.68 (0.23, 2.03)	0.489	0.90 (0.16, 5.12)	0.906	0.86 (0.14, 5.21)	0.866
MTBE	0.96 (0.59, 1.57)	0.873	0.98 (0.48, 1.99)	0.956	0.87 (0.41, 1.86)	0.712
o-Xylene	0.68 (0.20, 2.27)	0.524	1.04 (0.22, 5.06)	0.956	1.04 (0.19, 5.55)	0.964

Model 1 was adjusted for gender and age. Model 2 was additionally adjusted for race, FIPR, BMI, marital status, drinking status, smoking status, and physical activity level. Model 3 was additionally adjusted for hypertension, diabetes, hyperlipidemia, and CKD. FIPR, family income-to-poverty ratio; BMI, body mass index; CKD, chronic kidney disease.

## Data Availability

Data will be made available on request.

## Supplementary Materials

The following supporting information can be downloaded at: https://www.mdpi.com/article/10.3390/toxics12110772/s1, Figure S1: Evaluation criteria for clustering; Figure S2: Multi-variate adjusted odds ratios (95% CIs) of hyperuricemia in relation to the multiple VOCs co-exposure clusters in subgroups of participants in NHANES 2003-2012; Table S1: URLs of laboratory measurement of VOCs from the NHANES survey; Table S2: Eigenvalues of the covariance matrix of VOCs; Table S3: Distributions of eight blood VOCs pre-processed by ln-transformation and standardization; Table S4: The differences in the original concentrations of eight VOCs in four clusters; Table S5: Baseline characteristics of all participants in the NHANES 2003-2012; Table S6: Multi-variate adjusted odds ratios (95% CIs) of hyperuricemia in relation to the multiple VOCs co-exposure clusters among participants without medication use that could potentially affect UA metabolism; Table S7: Multi-variate adjusted odds ratios (95% CIs) of hyperuricemia in relation to the multiple VOCs co-exposure clusters after excluding participants with at least three of the four chronic diseases.

## Abbreviations

ACR, albumin-to-creatinine ratio; BMI, body mass index; CIs, confidence intervals; CKD, chronic kidney disease; CYP, cytochrome P450; DBPs, disinfection by-products; eGFR, estimated glomerular filtration rate; FIPR, family income-to-poverty ratio; IQR, interquartile range; MTBE, methyl tertiary-butyl ether; NHANES, National Health and Nutrition Examination Survey; ORs, odds ratios; RCS, restricted cubic spline; SUA, serum uric acid; UA, uric acid; VOC, volatile organic compound; VOCs, volatile organic compounds; XO, xanthine oxidase.
