# Supplementary material for: Assessing the Co-Exposure Patterns of Volatile Organic Compounds and the Risk of Hyperuricemia: An Analysis of the National Health and Nutrition Examination Survey 2003–2012
Source: Toxics. 2024 Oct 24;12(11):772. doi: 10.3390/toxics12110772 (PMC11598210; doi:10.3390/toxics12110772)
Supplement: Supplementary file 1 [file toxics-12-00772-s001.zip › Supplementary Figure S2.pdf]

Subgroup	Levels	OR(95%CI)	Forest plot	P value	P for interaction
Gender					0.992
Male	Cluster 1	1.00(ref)			
	Cluster 2	1.40(0.93,2.12)		0.107	
	Cluster 3	1.43(0.82,2.50)		0.201	
	Cluster 4	1.15(0.81,1.61)		0.428	
Female	Cluster 1	1.00(ref)			
	Cluster 2	1.30(0.78,2.14)		0.305	
	Cluster 3	1.21(0.67,2.19)		0.524	
	Cluster 4	1.06(0.69,1.63)		0.79	
Age					0.012
<60	Cluster 1	1.00(ref)			
	Cluster 2	1.48(1.08,2.02)		0.015	
	Cluster 3	1.91(1.15,3.18)		0.013	
	Cluster 4	1.23(0.88,1.71)		0.22	
≥60	Cluster 1	1.00(ref)			
	Cluster 2	1.13(0.73,1.74)		0.581	
	Cluster 3	0.57(0.30,1.10)		0.092	
	Cluster 4	1.13(0.66,1.95)		0.642	
BMI					0.076
<25	Cluster 1	1.00(ref)			
	Cluster 2	1.97(1.01, 3.84)		0.046	
	Cluster 3	2.74(1.15, 6.55)		0.024	
	Cluster 4	2.22(1.17, 4.19)		0.015	
≥25	Cluster 1	1.00(ref)			
	Cluster 2	1.28(0.97,1.69)		0.079	
	Cluster 3	1.20(0.74,1.95)		0.452	
	Cluster 4	1.03(0.79,1.35)		0.812	

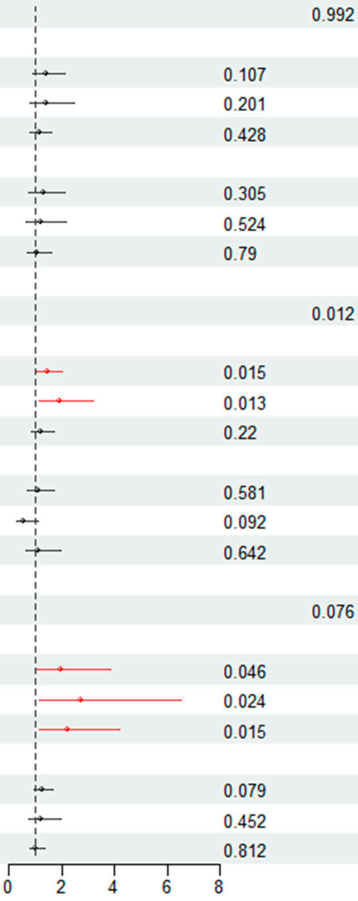

Supplementary Figure S2. Multi-variate adjusted odds ratios (95% CIs) of hyperuricemia in relation to the multiple VOCs co-exposure clusters in subgroups of participants in NHANES 2003-2012. Model 1 was adjusted for gender and age. Model 2 was additionally adjusted for race, FIPR, BMI, marital status, drinking status, smoking status, physical activity level. Model 3 was additionally adjusted for hypertension, diabetes, hyperlipidemia, and CKD. FIPR, family income-to-poverty ratio; BMI, body mass index; CKD, chronic kidney disease.
