# Supplementary material for: Assessing the Co-Exposure Patterns of Volatile Organic Compounds and the Risk of Hyperuricemia: An Analysis of the National Health and Nutrition Examination Survey 2003–2012
Source: Toxics. 2024 Oct 24;12(11):772. doi: 10.3390/toxics12110772 (PMC11598210; doi:10.3390/toxics12110772)
Supplement: Supplementary file 1 [file toxics-12-00772-s001.zip › Supplementary Table S1.pdf]

Supplementary Table S1. URLs of laboratory measurement of VOCs from the NHANES survey.

Laboratory measurement	Years	URLs
Benzene<br>1,4-Dichlorobenzene<br>Ethylbenzene<br>o-Xylene	2003–2004	https://wwwn.cdc.gov/Nchs/Nhanes/2003-2004/L04VOC_C.htm
	2005–2006	https://wwwn.cdc.gov/Nchs/Nhanes/2005-2006/VOCWB_D.htm
	2007–2008	https://wwwn.cdc.gov/Nchs/Nhanes/2007-2008/VOCWB_E.htm
	2009–2010	https://wwwn.cdc.gov/Nchs/Nhanes/2009-2010/VOCWB_F.htm
	2011–2012	https://wwwn.cdc.gov/Nchs/Nhanes/2011-2012/VOCWB_G.htm
Bromodichloromethane<br>Chloroform<br>Dibromochloromethane<br>MTBE	2003–2004	https://wwwn.cdc.gov/Nchs/Nhanes/2003-2004/L04VOC_C.htm
	2005–2006	https://wwwn.cdc.gov/Nchs/Nhanes/2005-2006/VOCWB_D.htm
	2007–2008	https://wwwn.cdc.gov/Nchs/Nhanes/2007-2008/VOCMWB_E.htm
	2009–2010	https://wwwn.cdc.gov/Nchs/Nhanes/2009-2010/VOCMWB_F.htm
	2011–2012	https://wwwn.cdc.gov/Nchs/Nhanes/2011-2012/VOCMWB_G.htm
