# Supplementary material for: Assessing the Co-Exposure Patterns of Volatile Organic Compounds and the Risk of Hyperuricemia: An Analysis of the National Health and Nutrition Examination Survey 2003–2012
Source: Toxics. 2024 Oct 24;12(11):772. doi: 10.3390/toxics12110772 (PMC11598210; doi:10.3390/toxics12110772)
Supplement: Supplementary file 1 [file toxics-12-00772-s001.zip › Supplementary Table S2.pdf]

Supplementary Table S2. Eigenvalues of the covariance matrix of VOCs.

	Eigenvalues	Proportion of Variance	Cumulative Proportion
1	2.466	0.308	0.308
2	2.094	0.262	0.570
3	1.146	0.143	0.713
4	0.866	0.108	0.822
5	0.730	0.091	0.913
6	0.492	0.062	0.974
7	0.128	0.016	0.990
