# Supplementary material for: Assessing the Co-Exposure Patterns of Volatile Organic Compounds and the Risk of Hyperuricemia: An Analysis of the National Health and Nutrition Examination Survey 2003–2012
Source: Toxics. 2024 Oct 24;12(11):772. doi: 10.3390/toxics12110772 (PMC11598210; doi:10.3390/toxics12110772)
Supplement: Supplementary file 1 [file toxics-12-00772-s001.zip › Supplementary Table S3.pdf]

Supplementary Table S3. Distributions of eight blood VOCs pre-processed by ln-transformation and standardization.

| Variables | Min | 25th percentile | Median | 75th percentile | Max |
|---|---|---|---|---|---|
| Benzene | -0.648 | -0.648 | -0.648 | 0.394 | 4.223 |
| Bromodichloromethane | -2.221 | -0.904 | -0.046 | 0.729 | 3.595 |
| Chloroform | -1.740 | -0.703 | 0.004 | 0.688 | 7.086 |
| Dibromochloromethane | -1.899 | -0.831 | -0.325 | 0.624 | 5.136 |
| 1,4-Dichlorobenzene | -0.992 | -0.992 | -0.317 | 0.427 | 4.484 |
| Ethylbenzene | -0.785 | -0.785 | -0.292 | 0.570 | 7.172 |
| MTBE | -2.021 | -0.678 | -0.678 | 0.387 | 5.124 |
| o-Xylene | -0.883 | -0.883 | -0.103 | 0.506 | 7.986 |
