# Supplementary material for: Assessing the Co-Exposure Patterns of Volatile Organic Compounds and the Risk of Hyperuricemia: An Analysis of the National Health and Nutrition Examination Survey 2003–2012
Source: Toxics. 2024 Oct 24;12(11):772. doi: 10.3390/toxics12110772 (PMC11598210; doi:10.3390/toxics12110772)
Supplement: Supplementary file 1 [file toxics-12-00772-s001.zip › Supplementary Table S4.pdf]

Supplementary Table S4. The differences in the original concentrations of eight VOCs in four clusters.

Variables	Cluster 1	Cluster 2	Cluster 3	Cluster 4	P value
Benzene (ng/ml)	0.02 (0.02, 0.02)	0.03 (0.02, 0.04)	0.19 (0.11, 0.31)	0.02 (0.02, 0.02)	< 0.0001
Bromodichloromethane (pg/ml)	0.77 (0.44, 1.40)	1.60 (0.81, 2.80)	1.37 (0.44, 3.10)	5.06 (3.40, 8.42)	< 0.0001
Chloroform (pg/ml)	5.23 (2.85, 9.89)	11.00 (6.40, 21.00)	8.70 (4.20, 17.00)	16.10 (8.99, 29.13)	< 0.0001
Dibromochloromethane (pg/ml)	0.44 (0.44, 0.77)	0.44 (0.44, 1.10)	0.44 (0.44, 1.60)	2.90 (1.63, 5.70)	< 0.0001
1,4-Dichlorobenzene (ng/ml)	0.08 (0.03, 0.21)	0.15 (0.08, 0.52)	0.08 (0.04, 0.24)	0.08 (0.03, 0.29)	< 0.0001
Ethylbenzene (ng/ml)	0.02 (0.02, 0.03)	0.03 (0.02, 0.05)	0.10 (0.07, 0.14)	0.02 (0.02, 0.03)	< 0.0001
MTBE (pg/ml)	0.99 (0.99, 1.80)	31.00 (11.00, 78.00)	0.99 (0.99, 3.60)	0.99 (0.99, 2.24)	< 0.0001
o-Xylene (ng/ml)	0.02 (0.02, 0.03)	0.03 (0.03, 0.05)	0.06 (0.05, 0.09)	0.02 (0.02, 0.03)	< 0.0001

Data are presented as the median (interquartile spacing).
