# Supplementary material for: Assessing the Co-Exposure Patterns of Volatile Organic Compounds and the Risk of Hyperuricemia: An Analysis of the National Health and Nutrition Examination Survey 2003–2012
Source: Toxics. 2024 Oct 24;12(11):772. doi: 10.3390/toxics12110772 (PMC11598210; doi:10.3390/toxics12110772)
Supplement: Supplementary file 1 [file toxics-12-00772-s001.zip › Supplementary Table S5.pdf]

Supplementary Table S5. Baseline characteristics of all participants in the NHANES 2003-2012.

| Characteristics | 2003-2004 | 2005-2006 | 2007-2008 | 2009-2010 | 2011-2012 | P value |
|---|---|---|---|---|---|---|
| n(%) | 984(13.14) | 1773(23.67) | 1366(18.24) | 1880(25.1) | 1487(19.85) | |
| Gender | | | | | | 0.38 |
| Male | 508(50.26) | 887(48.42) | 665(46.49) | 924(49.60) | 761(49.69) | |
| Female | 476(49.74) | 886(51.58) | 701(53.51) | 956(50.40) | 726(50.31) | |
| Age(years) | 41(30,49) | 45(34,59) | 46 (33,58) | 47(34,60) | 46(32,60) | < 0.0001 |
| Race/Ethnicity | | | | | | 0.8 |
| Non-Hispanic White | 508(70.06) | 899(72.68) | 589(66.41) | 864(65.70) | 574(67.29) | |
| Non-Hispanic Black | 220(11.53) | 413(10.98) | 330(13.52) | 341(11.72) | 367(11.39) | |
| Mexican American | 163(7.71) | 335(7.27) | 254(9.50) | 343(8.48) | 125(6.83) | |
| Other Hispanic | 37(4.06) | 54(3.52) | 144(5.35) | 227(6.27) | 164(7.39) | |
| Other Race | 56(6.63) | 72(5.54) | 49(5.22) | 105(7.83) | 257(7.10) | |
| FIPR | | | | | | 0.001 |
| <1.0 | 159(11.55) | 279(10.30) | 237(11.99) | 373(13.95) | 321(17.67) | |
| ≥1.0 | 784(88.45) | 1419(89.70) | 1015(88.01) | 1333(86.05) | 1035(82.33) | |
| BMI(kg/m²) | 26.84(23.39,31.06) | 27.57(24.11,32.00) | 27.52(24.12,31.57) | 27.62(24.11,32.33) | 27.40(23.90, 32.30) | < 0.001 |
| Marital status | | | | | | 0.004 |
| Married | 512(56.42) | 973(59.76) | 714(55.83) | 969(55.38) | 716(51.19) | |
| Living with partner | 87(7.85) | 131(7.88) | 97(7.10) | 150(7.18) | 116(8.79) | |
| Widowed | 20(1.92) | 165(6.18) | 119(5.75) | 174(6.85) | 109(5.50) | |
| Divorced | 92(9.30) | 167(8.90) | 159(10.43) | 215(10.60) | 152(9.93) | |
| Separated | 33(2.37) | 48(2.24) | 43(2.28) | 75(2.94) | 56(2.30) | |
| Never married | 239(22.14) | 288(15.04) | 234(18.61) | 297(17.06) | 336(22.30) | |
| Drinking status | | | | | | 0.42 |
| Never drinker | 99(9.03) | 210(11.08) | 178(11.88) | 220(10.14) | 186(10.79) | |
| Former drinker | 118(12.78) | 360(17.37) | 266(16.58) | 284(15.39) | 230(14.21) | |
| Mild drinker | 284(34.77) | 529(34.30) | 380(34.77) | 534(33.45) | 460(38.34) | |
| Moderate drinker | 161(18.62) | 250(16.95) | 169(14.85) | 238(17.04) | 186(14.91) | |
| Heavy drinker | 222(24.81) | 299(20.29) | 265(21.91) | 391(23.98) | 258(21.75) | |
| Smoking status | | | | | | 0.11 |
| Never smoker | 519(50.96) | 892(49.79) | 709(53.72) | 1012(55.31) | 829(55.40) | |
| Former smoker | 185(22.35) | 449(25.29) | 334(23.27) | 460(23.55) | 366(24.80) | |
| Current smoker | 280(26.70) | 431(24.92) | 322(23.02) | 408(21.14) | 292(19.79) | |
| Physical activity level | | | | | | < 0.0001 |
| High | 188(23.57) | 349(27.63) | 641(68.69) | 912(68.40) | 783(73.12) | |
| Moderate | 181(25.35) | 305(23.47) | 133(14.11) | 206(15.59) | 153(11.62) | |
| Insufficient | 414(51.08) | 675(48.89) | 170(17.19) | 233(16.01) | 186(15.26) | |
| Hypertension | | | | | | 0.002 |
| No | 702(72.12) | 1047(63.89) | 800(64.27) | 1084(64.21) | 867(62.84) | |
| Yes | 281(27.88) | 725(36.11) | 566(35.73) | 795(35.79) | 620(37.16) | |
| Diabetes | | | | | | < 0.0001 |
| No | 938(95.77) | 1558(90.83) | 1186(91.13) | 1646(91.78) | 1284(88.86) | |
| Yes | 46(4.23) | 215(9.17) | 180(8.87) | 234(8.22) | 203(11.14) | |
| Hyperlipidemia | | | | | | 0.22 |

| | | | | | | |
|---|---|---|---|---|---|---|
| No | 336(33.01) | 506(27.87) | 349(28.11) | 462(26.37) | 424(28.88) | |
| Yes | 648(66.99) | 1267(72.13) | 1017(71.89) | 1418(73.63) | 1063(71.12) | |
| CKD | | | | | | < 0.0001 |
| No | 893(92.55) | 1411(85.09) | 1080(85.78) | 1503(85.44) | 1221(86.80) | |
| Yes | 79(7.45) | 339(14.91) | 268(14.22) | 364(14.56) | 257(13.20) | |

FIPR, family income-poverty ratio; BMI, body mass index; CKD, chronic kidney disease.

P values were calculated using the Rao-Scott chi-square test for categorical variables and the Kruskal-Wallis test for continuous variables.
