# Supplementary material for: Assessing the Co-Exposure Patterns of Volatile Organic Compounds and the Risk of Hyperuricemia: An Analysis of the National Health and Nutrition Examination Survey 2003–2012
Source: Toxics. 2024 Oct 24;12(11):772. doi: 10.3390/toxics12110772 (PMC11598210; doi:10.3390/toxics12110772)
Supplement: Supplementary file 1 [file toxics-12-00772-s001.zip › Supplementary Table S6.pdf]

Supplementary Table S6. Multi-variate adjusted odds ratios (95% CIs) of hyperuricemia in relation to the multiple VOCs co-exposure clusters among participants without medication use that could potentially affect UA metabolism.

Variables	Model 1		Model 2		Model 3	
	OR (95%CI)	P value	OR (95%CI)	P value	OR (95%CI)	P value
Cluster 1	1.00(ref)		1.00(ref)		1.00(ref)	
Cluster 2	1.26(1.01,1.58)	0.043	1.48(1.13,1.95)	0.006	1.45(1.09,1.92)	0.011
Cluster 3	1.13(0.87,1.47)	0.359	1.54(1.00,2.37)	0.049	1.54(1.01,2.36)	0.047
Cluster 4	0.93(0.73,1.19)	0.562	1.11(0.83,1.48)	0.482	1.12(0.83,1.50)	0.457

Model 1 was adjusted for gender and age. Model 2 was additionally adjusted for race, FIPR, BMI, marital status, drinking status, smoking status, physical activity level. Model 3 was additionally adjusted for hypertension, diabetes, hyperlipidemia, and CKD. FIPR, family income-to-poverty ratio; BMI, body mass index; CKD, chronic kidney disease.
