# Supplementary material for: Assessing the Co-Exposure Patterns of Volatile Organic Compounds and the Risk of Hyperuricemia: An Analysis of the National Health and Nutrition Examination Survey 2003–2012
Source: Toxics. 2024 Oct 24;12(11):772. doi: 10.3390/toxics12110772 (PMC11598210; doi:10.3390/toxics12110772)
Supplement: Supplementary file 1 [file toxics-12-00772-s001.zip › Supplementary Table S7.pdf]

Supplementary Table S7. Multi-variate adjusted odds ratios (95% CIs) of hyperuricemia in relation to the multiple VOCs co-exposure clusters after excluding participants with at least three of the four chronic diseases.

Variables	Model 1		Model 2		Model 3	
	OR (95%CI)	P value	OR (95%CI)	P value	OR (95%CI)	P value
Cluster 1	1.00(ref)		1.00(ref)		1.00(ref)	
Cluster 2	1.21(0.96,1.52)	0.101	1.45(1.10,1.90)	0.040	1.37(1.02,1.85)	0.039
Cluster 3	1.15(0.87,1.53)	0.322	1.58(0.99,2.52)	0.111	1.54(0.96,2.47)	0.071
Cluster 4	1.03(0.80,1.33)	0.803	1.22(0.90,1.65)	0.068	1.19(0.88,1.63)	0.254

Model 1 was adjusted for gender and age. Model 2 was additionally adjusted for race, FIPR, BMI, marital status, drinking status, smoking status, physical activity level. Model 3 was additionally adjusted for hypertension, diabetes, hyperlipidemia, and CKD. FIPR, family income-to-poverty ratio; BMI, body mass index; CKD, chronic kidney disease.
