# Supplementary figures and images for: Assessing the Co-Exposure Patterns of Volatile Organic Compounds and the Risk of Hyperuricemia: An Analysis of the National Health and Nutrition Examination Survey 2003–2012
Source: Toxics. 2024 Oct 24;12(11):772. doi: 10.3390/toxics12110772 (PMC11598210; doi:10.3390/toxics12110772)

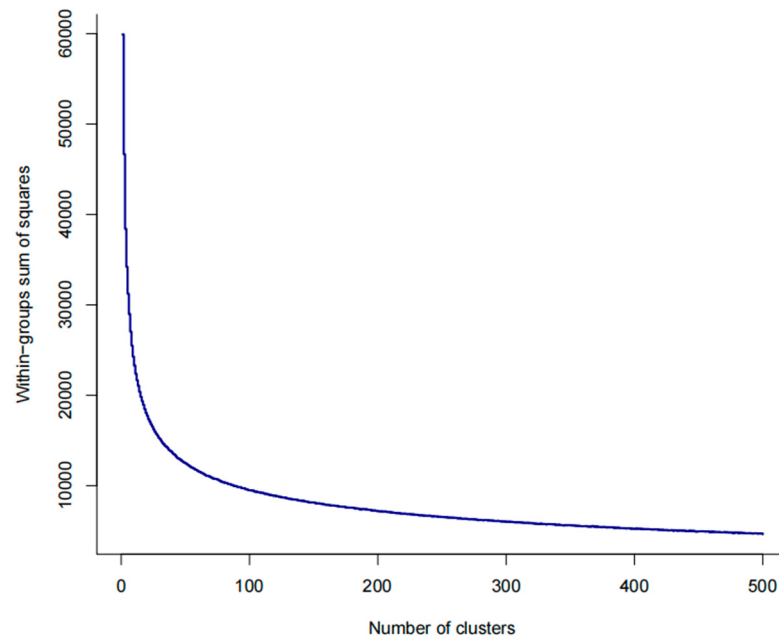

Supplementary Figure S1. Evaluation criteria for clustering.

Supplement: Supplementary file 1 [file toxics-12-00772-s001.zip › Supplementary Figure S1.pdf]
